# Relationship of Perceived Social Support with Mental Health in Older Caregivers

**DOI:** 10.3390/ijerph17113886

**Published:** 2020-05-30

**Authors:** Laura Muñoz-Bermejo, José Carmelo Adsuar, Salvador Postigo-Mota, Inés Casado-Verdejo, Claudia Mara de Melo-Tavares, Miguel Ángel Garcia-Gordillo, Jorge Pérez-Gómez, Jorge Carlos-Vivas

**Affiliations:** 1 Social Impact and Innovation in Health (InHEALTH), University of Extremadura, 10003 Cáceres, Spain; 2 Health, Economy, Motricity and Education Research Group (HEME), Faculty of Sport Sciences, University of Extremadura, 10003 Cáceres, Spain; jorgepg100@gmail.com (J.P.-G.); jorge.carlosvivas@gmail.com (J.C.-V.); 3 Department of Nursing, Faculty of Medicine, University of Extremadura, 06006 Badajoz, Spain; spostigo@unex.es; 4 Department of Nursing and Physiotherapy of Faculty of Health Sciences, University of León, 24071 León, Spain; ines.casado@unileon.es; 5 Department of Maternal, Child and Psychiatric Nursing, University Federal Fluminense/UFF, Rio de Janeiro (RJ) 24020-140, Brasil; claudiamarauff@gmail.com; 6 Facultad de Administración y Negocios, Universidad Autónoma de Chile, sede Talca 3467987, Chile; miguelgarciagordillo@gmail.com

**Keywords:** health surveys, aged, caregivers, mental health, stress, psychological, social support

## Abstract

Background: Elderly caregivers present increased physical and mental health problems. These factors can lead to a lack of autonomy and a need for social support. This study aims to analyse the relationships between perceived social support and mental health status in elderly caregivers aged 65 and older. Methods: a cross-sectional study based on data from the Spanish National Health Survey (ENSE-17) carried out on 7023 people. The study population was restricted to 431 caregivers aged ≥65 years. A study of the correlation between the mental health state and the perceived social support was carried out. Both variables were related to the sex of the caregiver. Results: Perceived social support by older caregivers is significantly related to mental health (*p* = 0.001), and stress (*p* < 0.001). Also, there is a significant relationship between perceived social support and mental well-being (*p* = 0.001), self-esteem (*p* = 0.005) and stress (*p* = 0.001) in older women caregivers. Conclusions: Older caregivers have adequate mental well-being and perceive high social support. Perceived social support can contribute to improving the mental well-being of older caregivers.

## 1. Introduction

Population ageing represents one of the greatest challenges among the developments resulting from demographic change in Western societies. As the population ages, the demand for informal caregiving by family members is constantly increasing [1]. The availability of potential caregivers in western countries is currently decreasing due to several demographic and socio-structural changes, such as lower fertility, increased mobility, and higher participation of women in the labour market [2]. The family is the main provider of care for dependent older people, and the spouse is the person who frequently takes the role of the main caregiver [3]. In many cases, adult children are providing care to their elderly parents, resulting in additional stress, which puts the caregivers’ mental health at risk as well [4].

According to the Survey of Living Conditions in Spain (ECV-2018) [5] and The English Longitudinal Study of Ageing (ELSA-2002-16) [6], the prevalence of caring for dependents are 11%−13% and 14%−17% among older men and women, respectively. The increase of men in care supposes a new trend in informal elderly care. Also, older people who are dedicated to caring are involved in performing their function at a high hourly rate. Specifically, more than two-thirds of these people spend more than 20 h a week on caring [7].

The caregiving role performance by older people requires a dedication to which their capacities may be diminished. The extra effort could influence the emergence of health problems in the caregivers [8].

The care of dependent people is associated with negative physical and mental health consequences for family caregivers [9,10,11]. The emotional and psychological consequences of caregiving are primarily represented by subjective burden [12], anxiety, depressive symptoms [13,14,15], higher levels of stress [16], lower levels of subjective well-being and self-efficacy [17], and low self-perceived quality of life [18,19]. Prevalence studies have shown that 40.2% of elderly relative caregivers present depressive symptoms [14].

To establish environments that keep caregivers in good health and preserve adequate caregiving skills, evidence is needed on the negative effects of health care. The health consequences of caregiving can be influenced by individual and contextual factors like age and sex, the duration of caregiving as well as support through family networks or community programs [20]. Several studies have supported the role of social support in protecting and maintaining physical and psychological health [21,22].

Social support in caregivers can be measured by the perceived social support and the social support received. Perceived social support refers to the availability of help when needed, the assessment of its relevance and/or quality of such assistance. Support received refers to the origin and frequency of specific help [23].

The relationships between social support and symptoms associated with the caregiver’s subjective burden depend on whether social support is measured as perceived or received. There is a relationship between perceived social support and the subjective burden. In contrast, there is no relationship between the support received and the subjective burden [24]. In this context, social support perceived as adequate or positive may be related to a less stressful situation and may favour the emotional well-being of older caregivers. Thus, emotional problems arising from the subjective burden on caregivers should be addressed as an objective of intervention by increasing social support [25]. However, to the best of our knowledge, no publication has studied the relationship between social support on mental well-being in older caregivers, previously.

Therefore, this study aims to analyse the relationship between perceived social support and mental health in caregivers aged 65 and older. If a direct correlation between perceived social support and mental health status were shown, it could make visible the need to develop strategies that implement the resources to help caregivers. Thus, preventing the emergence of emotional disturbances that worsen the mental health status of older caregivers.

## 2. Experimental Section

### 2.1. Study Design

A descriptive correlation study was carried out, taking as the primary data source the results of the Spanish National Health Survey (ENS) 2017 [9]. The survey is aimed at the group of persons who reside in primary family dwellings. If the dwelling includes two or more families, the study extends to all of them, but a survey will be carried out for each family. Stratified tri-stage sampling was used. The information collection period extends over one year, from October 2016 to October 2017, to collect data that may be affected by seasonality.

### 2.2. Sample Size Calculation

A sample size of 97 achieves 90% power to detect a difference of −0.31 between the null hypothesis correlation of 0.09 and the alternative hypothesis correlation of 0.4 using a two-sided hypothesis test with a significance level of 0.05 [26].

### 2.3. Ethical Approval

Files for public use are not considered confidential, following Regulation (EU) 2016/679 of the European Parliament and of the Council of 27 April 2016 on the protection of individuals concerning the processing of personal data and on the free movement of such data (which entered into force on 25 May 2016 and has been compulsory since 25 May 2018). Data protection principles do not need to be applied to anonymous information (i.e., information related to an identifiable natural person, nor to data of subject that is not, or is no longer, identifiable). Consequently, the Regulation does not affect the processing of information published by ENSE. Even for statistical or research purposes, its use does not require the approval of an accredited ethics committee.

### 2.4. Participants

We interviewed a total of 7023 participants aged 65 years or older. To be included in this analysis, participants need to meet the following inclusion criteria: (1) primary caregivers and (2) persons aged 65 years or older. After applying eligibility criteria, only 494 persons were considered for this study. Subsequently, persons who did not respond to survey items referring to mental health or perceived social support were excluded (*n* = 63). Therefore, the final sample was made up of 431 elderly caregivers (257 women and 174 men).

### 2.5. Measures and Procedures

A descriptive analysis was made of all sociodemographic factors, mental health characteristics and perceived social support.

Sociodemographic data. Information regarding age, sex and educational level of the participants was selected. Hours spent on care were also included. The variable “hours dedicated to caring” measures the total hourly burden of caring for persons per week. Possible responses were (a) less than 10 h per week, (b) 10 h or more per week but less than 20 h, and (c) 20 h or more per week.

Relationship to the caregiver. The kinship to caregiver variable is a dichotomous variable, where the possible choices are relatives or other persons.

Mental health status. It was measured by the General Health Questionnaire (GHQ-12), whose overall score provides an assessment of the psychological health of a population [27]. The GHQ-12 is made up of 12 items with answers graduating from 0 to 3, in which the person is asked whether they have recently experienced a series of symptoms or a particular behaviour. According to this method, the maximum value that can be obtained on the scale is 36 points, and the minimum is zero points. The total score is obtained by a simple summation of the scores on each of the items. The score, therefore, ranges from 0−36 points, from best to worst mental health. The optimal cut-off point is 2/3 of the maximum score. Scores of 12 or higher indicate the possibility that the person is suffering from an emotional disorder [28]. The Spanish version of the GHQ-12 is valid and reliable, presenting a high internal consistency in the general population (α = 0.86) and also in the population over 65 years of age (α = 0.90) [29].

From the exploratory factor analysis, it is observed that much of the variability of the GHQ-12 items can be explained by bringing together the 12 items into 3 factors. The existence of three factors: successful coping, self-esteem and stress are described by different authors [30,31,32].

Through the factorial analysis forcing the grouping of three factors, it is observed that in Factor I, 6 items are gathered (1, 3, 4, 7, 8 and 12) and these are called “Successful Coping”. In Factor II, there are 4 items (6, 9, 10 and 11) and these are called “Self-esteem”. In Factor III, there are 3 items (2, 5 and 9) and these are called “Stress”. Every question assesses the severity of a mental problem in the last few weeks using a 4-point Likert scale (0 to 3). Positive items were corrected from 0 (always) to 3 (never) and negative items from 3 (always) to 0 (never). Note that Item 9 presents loads on two factors, positively on Factor II and negatively on Factor III. The minimum value that can be reached in Factor 1 is 0 and the maximum is 18. Factor II has a range of 0−12 and Factor III of 0−9. High scores indicate poorer health.

The external validity of the 3 factors are 0.82, 0.70 and 0.75 for Factors I, II and III (*p* < 0.001), respectively.

To calculate the value of each factor, a recoding has been done by grouping the answers to the questions of each factor.

Perceived Social Support. The Duke-UNC-11 Functional Social Support Questionnaire [33] was used to determine the perceived social support of older caregivers. This scale has 11 items on a Likert response scale from 1 (“much less than I want”) to 5 (“as much as I want”). The score ranges from 11 to 55 points. In the Spanish version, a cut-off point was chosen at the 15th percentile, corresponding to a score of <32. A score of 32 or more indicates standard support, while a score lower than 32 points indicates low perceived social support. The Spanish version of this questionnaire is valid and reliable [34], showing excellent internal consistency in the Spanish population (α = 0.90).

### 2.6. Statistical Analysis

Statistical analysis was performed using the software IBM SPSS© version 22 (IBM, Armonk, NY, USA). Descriptive statistics are presented as mean and standard deviation, and median and interquartile range (IQR) for age, Goldberg’s general health status (GHQ-12) and DukeUNC-11 perceived social support. Percentages were obtained for Level of Studies, Relationships and Hours Spent Caring.

The Kolmogorov–Smirnov test was applied to check that the sample distribution data for every variable fit a normal distribution. Results showed that all variables did not fit a normal distribution. Thus, between-group differences were analysed using the Mann–Whitney U-test for continuous variables and the χ^2^ test for categorical variables. The significant level was set at a *p*-value of 0.05 for all tests. “Don’t know” and “no response” responses were considered missing data.

To evaluate possible differences between subgroups for each variable, we used the non-parametric Mann–Whitney U-test for two independent samples and the chi-square test for sex comparisons.

Subgroup comparison in GHQ-12, Factors I, II and III were also conducted according to Level of Studies, Relationships and Hours Spent Caring. Besides, to assess the existence of significant differences between the levels of GHQ-12, Factors I, II and III according to Level of Studies, Relationships and Hours Spent Caring, the Kruskal–Wallis non-parametric test was used for the variables GHQ-12, Factors I, II and III, using the different levels of the variables Level of Studies, Relationships and Hours Spent Caring. For posthoc analysis, the Mann–Whitney U-test was used for pairwise comparisons, with the corresponding Bonferroni correction to counteract the problem of multiple comparisons, establishing *p* ˂ 0.04.

Spearman’s Rho test was used to determine the correlation between GHQ-12 and Duke-UNC-11. Hypothesis contrasts were considered significant when *p* < 0.05. Bonferroni correction was applied for multiple comparisons, establishing *p* ˂ 0.01.

## 3. Results

Table 1 shows the descriptive statistics of the total sample (*n* = 431) and by sex subgroups: women (*n* = 257) and men (*n* = 174). The average age of the sample was 73.3 (6.5) years, with a median of 71 (10) years. 

For GHQ−12, mean and median values of less than 12 points were obtained. High scores indicate the possibility that the person is suffering from an emotional disorder. The results of Duke-UNC-11 means and medians were between 47 and 50 points. Values above 32 indicate low social support. 

Statistically significant differences (*p* ≤ 0.05) were found between the sex of older caregivers and the level of studies (*p* = < 0.001), relationship to the care recipient (*p* = < 0.001), hours spent caring (*p* = < 0.001), mental well-being (*p* = 0.001), successful coping (*p* = 0.006), self-esteem (*p* = 0.038), stress (*p* = < 0.001) and normal and low social support (*p* = < 0.001).

Table 2, Table 3, Table 4 and Table 5 show the differences between the subgroups of the level of studies, relationship with the recipient of care and hours dedicated to caring in the results of the level of GHQ-12 (Mental Health), Factor I (Successful Overcoming), Factor II (Self-esteem) and Factor III (Stress), respectively.

There is a continuous and progressive decrease in the mean values of GHQ-12, Factor I “Successful Coping”, Factor II “Self-esteem” and Factor III “Stress” as the level of education increases. It is noteworthy that this continuous decrease suffers a small peak increase in those caregivers who have an intermediate level of education based on “Vocational Training”. As the number of hours dedicated to caregiving increases, there is an increase in GHQ-12, Factor I, II and III values.

Besides, to evaluate the existence of significant differences between the levels of GHQ-12, Factors I, II and III, according to the level of studies, relationships and hours dedicated to caring, the Kruskal–Wallis non-parametric test was carried out using the different levels of the subgroups (SG) as factors. In all cases, *p* < 0.005.

Since in all four cases, the result of the Kruskal–Wallis test indicated the existence of significant differences in at least one of the medians, post hoc analyses were performed using the Mann–Whitney U-test for the variables GHQ-12, Factors I, II and III, to detect among which specific subgroups of study level, relationships and hours spent caring occurred these significant differences. In these analyses, Bonferroni corrections were applied to counteract the problem of multiple comparisons by obtaining *p* < 0.004.

The results of these post hoc analyses show that the significant differences in GHQ-12 are mainly among the lower education subgroups compared to those with university education and among the subgroups that spend less than 10 h per week in caregiving and those that spend more than 20 h per week. In the results for Factors I and III, significant differences are shown between elderly caregivers with intermediate education and those with higher education. In Factor II, there are significant differences between those with lower and higher studies. Also, significant differences are shown between those who spend less than 10 h per week on caregiving compared to those who spend more than 20 h a week on caregiving.

The Spearman correlation coefficient was calculated for GHQ-12, Factors I, II and III, according to the Duke-UNC-11 by the older caregivers in the sample. Besides, the calculation was made both generally for the sample as a whole and independently for the sex subgroup within the perceived social support (Table 6). A significant correlation was obtained for both the variable GHQ-12 (R = −0.153) and FIII (R = −0.199) as far as Duke-UNC-11—Perceived Social Support was concerned for the analysis of the total sample. Both values showed a direct negative correlation. Similarly, in the calculation of the respective correlation coefficients of the women in the sample, significant correlations were obtained with a value of R = −0.236 for the association between GHQ-12 and Duke-UNC-11, the value of R = −0.174 for the relationship between FII and Duke-UNC-11, and the value of R = −0.303 for the relationship between FIII and Duke-UNC-11.

## 4. Discussion

The main finding of our study is that perceived social support by older caregivers is related to mental health and stress levels. This relationship is significantly present in women, where it is also related to self-esteem.

These results are in line with previous studies [35,36] that highlights the importance of social support as a variable that moderates the negative impact on the caregiver’s health. Our results also show that women caregivers care for them report longer working hours, higher stress levels, and a greater decline in self-esteem and coping skills than men. Previous studies have also argued that women caregivers report a greater burden, as well as more stress and depression than men [37,38,39].

In this sense, the mental health of women caregivers is more impaired than that of male caregivers [40]. It may be due to their greater involvement in care in terms of both quantity and quality of care (type and manner of care). The greater involvement of women caregivers would, therefore, explain because the negative impact of caregiving is more intense for women than for men [41].

On the other hand, our results highlight that older caregivers perceive high social support and present a good state of mental health, low-stress levels and adequate levels of self-esteem and coping skills. As other research points out, informal social support provided by the family is beneficial in alleviating the burden on the caregiver and improving coping capacities [42,43]. Ongoing social support contributes to stable self-esteem [44], and the lack of social support in stressful situations has an impact on the emotional stability of the person who is seeking help [45].

As the age of caregivers increases, so does the risk of illness and autonomy limitations. In this sense, the personal effort of the caregivers is increased, and help may be needed [8,46].

Our results show no significant differences in the relationship between the caregiver and the care recipient. However, other studies indicate that bivariate analyses revealed that caring for primary relatives (i.e., spouse, parents, and children) was associated with greater self-reported stress than caring for other family members or non-family members (such as friends, neighbours, or co-workers) [47].

Another of the study findings is that older caregivers have a decrease in GHQ-12 (increased mental well-being) as their level of education increases and as their hours of caregiving decrease. The same is true for Factors I, II and III. In line with these results, other studies show there is evidence that both men and women are at high risk of deteriorating mental health when they perform a high number of hours of care for adult dependents [48]. The caregiver’s social supports can improve reduced mental health symptoms [49].

According to Survey of Health, Ageing and Retirement (SHARE, waves 1−8) and the English Longitudinal Study of Ageing (ELSA, wave 2−9), caring for a dependent person has negative effects on the caregiver’s health [4,6]. In such care situations, the social networks in the caregivers and recipients of care that are brought in can play an important role in addressing the challenges of the care situation [50]. Increased social contacts are associated with improved health and could, therefore, be essential in protecting older caregivers [51].

The main strengths of this study are the sample size and the fact that a nationally representative survey was used. The use of the National Health Survey provides representative data on the health of the elderly care population and the relationship between sociodemographic characteristics, perceived social support and mental health. These results make it possible to identify elderly caregivers as one of the most vulnerable groups in the population. However, like all studies, it also has some limitations: (1) GHQ-12 is a screening tool, more sensitive than specific, which can lead to an overestimation of mental health problems. It should be noted that false positives are more frequent in the population aged 65 years or older (population included in this study). (2) A control group of non-caregivers aged 65 and older was not included in the study, and (3) due to this being a cross-sectional and not an intervention study, it is not possible to establish cause–effect relationships.

### Implications of the Study

These findings lead to the need to include in care protocols for older people a specific assessment of perceived social supports and to establish strategies to meet their needs as caregivers. Actions of prevention, identification and attention to mental health problems should be considered as they could have an impact on the health of the caregiving population. Finally, the importance of strengthening and defending social welfare policies aimed at the comprehensive care of the elderly is highlighted, in which special attention and assessment of elderly caregivers, who due to circumstances are increasingly numerous in our society, must be included as a priority.

## 5. Conclusions

1. Older caregivers included in this study have high social support and good mental health. Self-esteem levels and coping skills are adequate, and the level of stress is low.
2. The results of this study suggest that there is a direct association between the level of studies of older caregivers and mental well-being. A higher level of studies in older caregivers is associated with greater mental well-being.
3. There can be a decline in the mental health of older caregivers if they spend more than 20 h a week caring.
4. The mental health of older caregivers could be related to perceived social support, especially in women.

## Figures and Tables

**Table 1:** Sociodemographic characteristics and dimensions-subscales of the Goldberg General Health Questionnaire GHQ-12 and Duke-UNC Functional Social Support Questionnaire in older caregivers. Spain, 2017.

Variables	Total Sample *n* = 431	Women *n* = 257	Men *n* = 174	*p*-Value
**Age (years)**				0.459 ^a^
Median (IQR)	71 (10)	71 (15)	71 (10)	
Mean (SD)	73.3 (6.5)	73.2 (6.3)	73.6 (6.9)	
Range	65−93	65−92	65−93	
**Level of Studies**				<0.001 ^b^
Cannot Read or Write	17 (3.9%)	14 (5.4%)	3 (1.7%)	
Primary School	233 (54.1%)	144 (56%)	89 (51.1%)	
Secondary School	96 (22.3%)	53 (20.6%)	43 (24.7%)	
Vocational Training	24 (5.6%)	15 (5.8%)	9 (5.2%)	
University Studies	61 (14.2%)	31 (12.1%)	30 (17.2)%	
**Relationship**				<0.001 ^b^
Family Members	401 (93%)	238 (92.6%)	163 (93.7%)	
Other People	30 (7%)	19 (7.4%)	11 (6.3%)	
**Hours Dedicated to Caring**				<0.001 ^b^
Less than 10 h per week	95 (22%)	50 (19.5%)	45 (25.9%)	
10 h or more a week, but less than 20 h	61 (14.2%)	25 (9.7%)	36 (20.7%)	
More than 20 h a week	275 (63.8%)	182 (70.8%)	93 (53.4%)	
**GHQ-12 (Mental Health)**				0.001 ^a^
Median (IQR)	11 (6)	11 (6)	9 (5)	
Mean (SD)	11.3 (5.1)	12 (5.3)	10.2 (4.6)	
Range	0−36	4−36	0−36	
**FI: Successful coping**				0.006 ^a^
Median (IQR)	6 (1)	6 (1)	6 (0)	
Mean (SD)	6,5 (1,9)	6,8 (2)	6,2 (1.7)	
Range	0−18	1−18	0−18	
**FII: Self-esteem**				0.038 ^a^
Median (IQR)	1 (3)	2 (3)	1 (3)	
Mean (SD)	1.7 (1.7)	1.8 (1.9)	1.5 (1.6)	
Range	0−9	0−9	0−9	
**FIII: Stress**				<0.001 ^a^
Median (IQR)	3 (3)	3 (4)	2 (2)	
Mean (SD)	3 (2.5)	3.3 (2.3)	2.5 (2.1)	
Range	0−9	0−9	0−9	
**Duke-UNC-11 (Perceived Social Support)**				0.314 ^a^
Median (IQR)	49 (9)	50 (12)	48.5 (10)	
Mean (SD)	47.2 (7.6)	47.3 (8.2)	47.1 (6.9)	
Range	17−55	17−55	20−55	
Normal Perceived Social Support	415 (96.3)	245 (95.3)	170 (97.7)	<0.001 ^b^
Low Perceived Social Support	16 (3.7)	12 (4.7)	4 (2.3)	<0.001 ^b^

The variables Level of Studies, Relationship and Hours Spent Caring, Normal Perceived Social Support and Low Perceived Social Support are presented as the total number of participants who fulfilled that condition (percentage for the entire sample). Goldberg’s general health status (GHQ-12): Points from 0 to 36 where 0—better mental health and 36—worse mental health; FI—Successful Coping: Points from 0 to 18 where 0—better successful coping and 18—worse successful coping; FII—Self-esteem: Points from 0 to 9 where 0—better self-esteem and 9—worse self-esteem; FIII—Stress: Points from 0 to 9 where 0—no stress and 9—intense stress; Duke-UNC-11: Scores between 11 and 55, a value ≥32 means normal perceived social support and a value ˂32 means low perceived social support. *p*-value: ^a^ *p*-value from U Mann–Whitney test and ^b^ *p*-value from x^2^ test.

**Table 2:** Comparison of GHQ-12 according to the level of studies, relationship with the care recipient and hours spent in care.

Variables	Levels		GHQ-12	SG	Means Diff	Medians Diff	*p* *	*p* **
Level of Studies	1	Median (IR) Mean (SD)	13 (9) 14.7 (7.0)	2 3 4 5	2.6 4.8 3.4 5.6	2 4 1.5 3.9	˂0.001	0.118 0.005 0.120 0.001
	2	Median (IR) Mean (SD)	11 (7) 12.1 (5.4)	1 3 4 5	−2.6 2.2 0.8 3	−2 2 3.1 3		0.118 ˂0.001 0.489 ˂0.001
	3	Median (IR) Mean (SD)	9 (5) 9.9 (4.0)	1 2 4 5	−4.8 −2.2 −1.4 0.8	−4 −2 −2.5 1		0.005 ˂0.001 0.221 0.198
	4	Median (IR) Mean (SD)	11.5 (6) 11.3 (4.8)	1 2 3 5	−3.4 −0.8 1.4 2.2	−1.5 0.5 2.5 3.5		0.120 0.489 0.221 0.048
	5	Median (IR) Mean (SD)	8 (6) 9.1 (4.0)	1 2 3 4	−5.6 −3 −0.8 −2.2	−5 −3 −1 −3.5		0.001 ˂0.001 0.198 0.048
Relationship	1	Median (IR) Mean (SD)	11 (6) 11.3 (5.2)	2	1	−0.5	NA	0.541
	2	Median (IR) Mean (SD)	11.5 (7) 10.3 (3.8)	1	−1	0.5		0.541
Hours Dedicated to Caring	1	Median (IR) Mean (SD)	10 (6) 9.9 (4.0)	2 3	−0.6 −2	0 −1	0.011	0.361 0.004
	2	Median (IR) Mean (SD)	10 (4.5) 10.5 (3.9)	1 3	0.6 −1.4	0 −1		0.361 0.144
	3	Median (IR) Mean (SD)	11 (7) 11.9 (5.6)	1 2	2 1.4	1 1		0.004 0.144

* Kruskal–Wallis global for *p* = 0.05, using as an answer, the Mental Health according to GHQ-12, and as factors, the Level of Studies: 1 (Cannot read or write), 2 (Primary School), 3 (Secondary School), 4 (Vocational Training) or 5 (University Studies); Relationship: 1 (Family members) or 2 (Other people); Hours Dedicated to Caring: 1 (Less than 10 h per week), 2 (10 h or more a week, but less than 20 h) or 3 (More than 20 h a week). ** Mann–Whitney post hoc U analysis, with Bonferroni correction factor having *p* = 0.004. GHQ-12 (values 0−36). SG: subgroups of Level of Studies (values 1−5), Relationship (1−2), Hours Dedicated to Caring (values 1−3). Mean Diff: the difference between the average values of GHQ−12 for each SG level. Median Diff: the difference between the mean values of GHQ−12 for each SG level. NA: not applicable.

**Table 3:** Comparison of Factor I “Successful Coping” according to the level of studies, relationship and hours dedicated to caring.

Variables	Levels		FI Successful Coping	SG	Means Diff	Medians Diff	*p* *	*p* **
Level of Studies	1	Median (IQR) Mean (SD)	6 (2.5) 7.4 (3.0)	2 3 4 5	0.6 1.3 0.6 1.5	0 0 0 0	˂0.001	0.777 0.056 0.988 0.091
	2	Median (IQR) Mean (SD)	6 (1) 6.8 (2.1)	1 3 4 5	−0.6 0.7 0.0 0.9	0 0 0 0		0.777 ˂0.001 0.723 0.004
	3	Median (RI) Media (DT)	6 (0) 6.1 (1.2)	1 2 4 5	−1.3 −0.7 −0.7 0.2	0 0 0 0		0.056 ˂0.001 0.009 0.687
	4	Median (RI) Media (DT)	6 (1.8) 6.8 (1.6)	1 2 3 5	−0.6 0.0 0.7 0.9	0 0 0 0		0.988 0.723 0.009 0.023
	5	Median (IQR) Mean (SD)	6 (0) 5.9 (1.5)	1 2 3 4	−1.5 −0.9 −0.2 −0.9	0 0 0 0		0.091 0.004 0.687 0.023
Relationship	1	Median (IQR) Mean (SD)	6(1) 6.6 (1.9)	2	0.5	0	NA	0.164
	2	Median (RI) Media (DT)	6 (0) 6.1 (1.1)	1	−0.5	0		0.164
Hours Dedicated to Caring	1	Median (RI) Median (DT)	6 (0) 6.1 (1.3)	2 3	−0.2 −0.7	0 0	0.028	0.222 0.010
	2	Median (IQR) Mean (SD)	6 (0) 6.3 (1.2)	1 3	0.2 −0,5	0 0		0.222 0.329
	3	Median (IQR) Mean (SD)	6 (1) 6.8 (2.1)	1 2	0.7 0.5	0 0		0.010 0.329

* Kruskal–Wallis global for *p* = 0.05, using as an answer, the Mental Health according to Factor I “Successful Coping”, and as factors, the Level of Studies: 1 (Cannot read or write), 2 (Primary School), 3 (Secondary School), 4 (Vocational Training) or 5 (University Studies); Relationship: 1 (Family members) or 2 (Other people); Hours Dedicated to Caring: 1 (Less than 10 h per week), 2 (10 h or more a week, but less than 20 h) or 3 (More than 20 h a week). ** Mann–Whitney post hoc U analysis with Bonferroni correction factor having *p* = 0.004. Factor I “Successful Coping” (values 0−18). SG: subgroups of Level of Studies (values 1−5), Relationship (1−2), Hours Dedicated to Caring (values 1−3). Mean Diff: the difference between the average values of Factor I “Successful Coping” for each SG level. Median Diff: the difference between the mean values of Factor I “Successful Coping” for each SG level. NA: not applicable.

**Table 4:** Comparison of Factor II “Self-esteem” according to the level of studies, relationship and hours dedicated to caring.

Variables	Levels		FII Self-esteem	SG	Means Diff	Medians Diff	*p* *	*p* **
Level of Studies	1	Median (IQR) Mean (SD)	3 (3) 3.1 (2.3)	2 3 4 5	1.1 1.8 1.6 2	1 2 2 3	˂0.001	0.045 0.001 0.018 ˂0.001
	2	Median (IQR) Mean (SD)	2 (3) 2.0 (1.8)	1 3 4 5	−1.1 0.7 0.5 0.9	−1 1 1 2		0.045 0.001 0.176 ˂0.001
	3	Median (RI) Median (DT)	1 (3) 1.3 (1.5)	1 2 4 5	−1.8 −0.7 −0.2 0.2	−2 −1 0 1		0.001 0.001 0.588 0.399
	4	Median (RI) Median (DT)	1 (3) 1.5 (1.7)	1 2 3 5	−1.6 −0.5 0.4 0.4	−2 −1 0 1		0.018 0.176 0.588 0.283
	5	Median (IQR) Mean (SD)	0 (2) 1.1 (1.3)	1 2 3 4	−2 −0.9 −0.2 −0.4	−3 −2 −1 −1		˂0.001 ˂0.001 0.399 0.283
Relationship	1	Median (IQR) Mean (SD)	1 (3) 2.6 (1.8)	2	1.1	0	NA	0.684
	2	Median (IQR) Mean (SD)	1 (3) 1.5 (1.4)	1	−1.1	0		0.684
Hours Dedicated to Caring	1	Median (IQR) Mean (SD)	1 (2) 1.3 (1.3)	2 3	−0.2 −0.6	0 −1	0.009	0.455 0.003
	2	Median (IQR) Mean (SD)	1 (3) 1.5 (1.5)	1 3	0.2 −0.4	0 −1		0.455 0.123
	3	Median (IQR) Mean (SD)	2 (3) 1.9 (1.9)	1 2	0.6 0.4	1 1		0.003 0.123

* Kruskal–Wallis global for *p* = 0.05, using as an answer, the Mental Health according to Factor II “Self-esteem”, and as factors, the Level of Studies: 1 (Cannot read or write), 2 (Primary School), 3 (Secondary School), 4 (Vocational Training) or 5 (University Studies); Relationship: 1 (Family members) or 2 (Other people); Hours Dedicated to Caring: 1 (Less than 10 h per week), 2 (10 h or more a week, but less than 20 h) or 3 (More than 20 h a week). ** Mann–Whitney post hoc U analysis with Bonferroni correction factor having *p* = 0.004. Factor II “Self-esteem” (values 0−12). SG: subgroups of Level of Studies (values 1−5), Relationship (1−2), Hours Dedicated to Caring (values 1−3). Mean Diff: the difference between the average values of Factor I “Self-esteem” for each SG level. Median Diff: the difference between the mean values of Factor I “Self-esteem” for each SG level. NA: not applicable.

**Table 5:** Comparison of Factor III “Stress” according to the level of studies, relationship and hours dedicated to caring.

Variables	Levels		FIII Stress	SG	Means Diff	Medians Diff	*p* *	*p* **
Level of Studies	1	Median (IQR) Mean (SD)	4 (4) 4.3 (2.8)	2 3 4 5	1 1.7 1.3 2.3	1 2 2 3	˂0.001	0.122 0.014 0.098 0.003
	2	Median (IQR) Mean (SD)	3 (3) 3.3 (2.2)	1 3 4 5	−1 0.7 0.3 1.3	−1 1 0 2		0.122 0.003 0.350 ˂0.001
	3	Median (RI) Median (DT)	2 (3) 2.6 (2.1)	1 2 4 5	−1.7 −0.7 −0.4 0.6	−2 −1 −1 1		0.014 0.003 0.485 0.084
	4	Median (RI) Median (DT)	3 (4) 3.0 (2.3)	1 2 3 5	−1.3 −0.3 0.4 1	−1 0 1 2		0.098 0.350 0.485 0.080
	5	Median (IQR) Mean (SD)	1 (3) 2.0 (2.0)	1 2 3 4	−2.3 −1.3 −0.6 −1	−3 −2 −1 −2		0.003 ˂0.001 0.084 0.080
Relationship	1	Median (IQR) Mean (SD)	3 (3) 3.0 (2.3)	2	0.4	0	NA	0.488
	2	Median (IQR) Mean (SD)	3 (4.3) 2.6 (2.1)	1	−0.4	0		0.488
Hours Dedicated to Caring	1	Median (IQR) Mean (SD)	3 (3) 2.6 (2.2)	2 3	−0.1 −0.6	0 0	0.082	0.634 0.036
	2	Median (IQR) Mean (SD)	3 (3) 2.7 (1.9)	1 3	0.1 −0.5	0 0		0.634 0.224
	3	Median (IQR) Mean (SD)	3 (4) 3.2 (2.3)	1 2	0.6 0.5	0 0		0.036 0.224

* Kruskal–Wallis global for *p* = 0.05, using as an answer, the Mental Health according to Factor III “Stress”, and as factors, the Level of Studies: 1 (Cannot read or write), 2 (Primary School), 3 (Secondary School), 4 (Vocational Training) or 5 (University Studies); Relationship: 1 (Family members) or 2 (Other people); Hours dedicated to caring: 1 (Less than 10 h per week), 2 (10 h or more a week, but less than 20 h) or 3 (More than 20 h a week). ** Mann–Whitney post hoc U analysis with Bonferroni correction factor having *p* = 0.004. Factor II “Stress” (values 0−9). SG: subgroups of Level of Studies (values 1−5), Relationship (1−2), Hours Dedicated to Caring (values 1−3). Mean Diff: the difference between the average values of Factor I “Stress” for each SG level. Median Diff: the difference between the mean values of Factor I “Stress” for each SG level. NA: not applicable.

**Table 6:** Relationship between the dimensions and subscales of the Goldberg General Health Questionnaire GHQ-12 and the Duke-UNC-11 Functional Social Support Questionnaire in older caregivers. Spain, 2017.

Duke UNC-11 (Perceived Social Support)						
Variables	Total Sample		Women		Men	
	Rho	*p*	Rho	*p*	Rho	*p*
**GHQ-12 (Mental Health)**	−0.153	0.001	−0.236	˂0.001	−0.060	0.434
**FI: Coping Capacity**	−0.112	0.020	−0.148	0.017	−0.084	0.272
**FII: Self-esteem**	−0.121	0.012	−0.174	0.005	−0.059	0.440
**FIII: Stress**	−0.199	˂0.001	−0.303	˂0.001	−0.067	0.381

Rho: Spearman correlation coefficient with the Bonferroni correction factor having *p* = 0.01.
